# Rapid detection of multidrug resistant tuberculosis in respiratory specimens at a tertiary care centre in south coastal Karnataka using Genotype MTBDR *plus* assay

**Published:** 2018-10

**Authors:** Vishnu Prasad Shenoy, Ajay Kumar, Kiran Chawla

**Affiliations:** Department of Microbiology, Kasturba Medical College, Manipal Academy of Higher Education, Manipal, Karnataka, India

**Keywords:** Genes, GenoType MTBDR *plus*, Mutations, Resistance, Tuberculosis

## Abstract

**Background and Objectives:**

Despite high prevalence of MDR-TB in India very limited information about MDR-TB and mutation patterns in *rpoB, inhA* and *katG* genes among MDR-isolates of *Mycobacterium tuberculosis* in south coastal Karnataka region is available; thus present study is an attempt to explore the extent of MDR-TB and mutation patterns prevalent among clinical isolates in this region using GenoType MTBDR *plus* assay.

**Materials and Methods:**

A total of 256 sputum samples from Pulmonary TB patients suspected of MDR-TB were tested by GenoType MTBDR *plus* as per manufacturer’s guidelines for detection of mutations conferring resistance to rifampicin and isoniazid. The results of GenoType MTBDR *plus* were recorded and analysed using SPSS version 22. For all analyses, a *p* value <0.05 was considered statistically significant.

**Results:**

Fifty (19.53%) isolates were found MDR, 32 (12.50%) isolates were found mono-resistant to isoniazid and 15 (5.86%) isolates were found mono-resistant to rifampicin. Eleven isolates (4.3%) were found NTM. Mutation in codon S531L, S315T1 and C15T were most common in *rpoB, katG* and *inhA* genes respectively. Unknown mutations were found in 50.77% (33/65), 3.66% (3/82) and 26.83% (22/82) isolates for *rpoB, katG* and *inhA* genes respectively. Hetero-resistance in MDR, rifampicin monoresistant and isoniazid monoresistant isolates was found to be 26% (13/50), 20% (3/15) and 34.37% (11/32) respectively.

**Conclusion:**

Mutation in codon S531L, S315T1 and C15T were most common mutations associated with MDR-TB. Further high number of isolates showed mutations in unknown regions and hetero-resistance thus more elaborate studies based on sequencing are desirable in this region.

## INTRODUCTION

Despite valiant efforts, tuberculosis (TB) remained a global concern with an annual incidence of 10.4 million new cases and accounting for 1.8 million deaths (1). Though TB is curable with appropriate treatment, emergence of drug resistance strains of *Mycobacterium tuberculosis* has complicated the situation of Global TB control program. In 2015, World Health organization (WHO) estimated 5, 80,000 new cases of drug resistant TB globally, 480,000 among them were mutli drug resistant tuberculosis (MDRTB) i.e. resistance to both isoniazid and rifampicin which are the most potent drugs in management of tuberculosis (1). India is the country with highest burden of TB; MDR TB is reported about 3% in new cases and 12–17% in retreatment cases (2). Due to complexity in assessing MDR TB the true global burden of MDR-TB remains underreported, especially in high burden countries like India where a large proportion of population is treated by private practitioners and majority of them not report to RNTCP. As per WHO report India along with China, Russian Federation, Indonesia and Nigeria accounted for more than 60% of the gap between MDR-TB detection and treatment (1). Rapid detection of MDR-TB is major intervention to reduce morbidity, mortality and ensure better management, thereby reducing economic cost, transmission of infection and emergence of extremely drug resistant strains (3). Conventional drug susceptibility testing on LJ media is considered gold standard for diagnosis of MDR TB, however it is time consuming and numerous problems are associated with the standardization of tests and stability of the drugs (4). Although introduction of broth based culture systems has improved the sensitivity and reduced the time required for DST testing as compared to solid culture methods but their contamination rate is very high, are expensive and requires particular expertise (5). The slow diagnosis of MDR-TB contributes in transmission of the disease to community and if not treated timely may lead to emergence of extensively drug resistant TB or totally drug resistant TB (6). In recent years several rapid molecular tests based on detection of mutations in the specific gene conferring drug resistance has been developed to address the challenges in detection of MDR-TB, of these tests GenoType MTBDR *plus* (Hain Lifescience, Germany) has shown promises to be an ideal rapid tool for early detection of MDR-TB and thus endorsed by WHO for detection of isoniazid and rifampicin resistance (7, 8). Geno-Type MTBDR *plus* is a line probe assay based on multiplexed polymerase chain reaction coupled with reverse hybridization on a nitrocellulose membrane targeting common mutations in *rpoB* gene (coding for β-subunit of RNA polymerase) for rifampicin resistance, *katG* gene (coding for catalase peroxidise) and *inhA* gene (coding for NADH enoyl ACP reductase) for high and low level isoniazid resistance respectively (9). Various meta-analysis on evaluation of performance of GenoType MTBDR *plus* reported excellent pooled sensitivity and specificity for detection of resistance to isoniazid, rifampicin and MDR (10, 11). Very limited information about MDR-TB and mutation patterns in *rpoB* gene, *inhA* gene and *katG* gene in MDR-isolates of *M. tuberculosis* in south coastal Karnataka region of India is available, thus in this study we made an attempt to explore the extent of MDR-TB and mutation patterns prevalent among clinical isolates of south coastal Karnataka using GenoType MTBDR *plus* assay..

## MATERIALS AND METHODS

Present study was carried out at a tertiary care hospital in south coastal Karnataka, India after obtaining approval from institutional research and ethical committee. A total of 256 sputum samples from Pulmonary TB patients suspected of MDR-TB (Patients failing first line regimen, all previously treated patients found smear positive at the end of extended intensive phase or late CAT-II, smear positive contacts of known MDR cases, new patient with doubtful history) were analyzed by GenoType MTBDR *plus* for detection of mutations conferring drug resistance from July 2011 to April 2017.

### Smear microscopy.

For all sputum samples AFB smears were prepared and were stained with Auramine O and counter stained with potassium permanganate and then were evaluated as per Revised National Tuberculosis Control Program (RNTCP) guidelines (12).

### Genotype MTBDR *plus* assay.

All samples were decontamination by N-acetyl-L-cysteine and sodium hydroxide (NALC-NaOH) method (13). All smear positive samples were subjected directly to Genotype MTBDR *plus* assay as per manufacturer’s instructions using *M. tuberculosis* H37Rv strain as internal quality control whereas all smear negative samples were cultured in liquid medium and were processed after obtaining growth.

### Statistical analysis.

The result of GenoType MTBDR *plus* assays and patients demographic details were recorded and analysed using SPSS version 22 (SPSS Inc., USA). Data were compared using Chi square test. For all analyses, a p value <0.05 was considered statistically significant.

## RESULTS

A total of 256 TB patients comprising of 34 (13.28%) treatment defaulters, 39 (15.23%) relapse cases, 7 (2.73%) treatment failure cases, 34 (13.28%) cases with history of TB, 112 (43.75%) cases on ATT treatment and 30 (11.72%) new cases with doubtful history. Average age of the patient was 43.38 years (SD: 14.64, range: 3–81 years). Two hundred thirty one samples (90.23%) were smear positive and 25 (9.77%) were smear negative. Fifty five (21.48%) patients were diabetic and 13 (5.09%) were co-infected with HIV.

On GenoType MTBDR *plus* analysis 148 (57.81%) isolates were found sensitive to both isoniazid and rifampicin. Fifty (19.53%) isolates were found MDR i.e. resistant to both isoniazid and rifampicin; Thirty two (12.50%) isolates were found mono-resistant to isoniazid and 15 (5.86%) isolates were found mono-resistant to rifampicin. Further in 11 (4.3%) smear positive isolates TUB band was absent thus were considered as Non Tuberculous Mycobacteria (NTM).

Among 65 rifampicin resistant isolates *rpo*BMUT3 band conferring mutation in S531L codon was present in 30 isolates (46.15%, 3/15 in Rif mono-resistant and 27/50 in MDR isolates). Difference of *rpo*B S531L mutations in MDR-isolates compared with rifampicin mono-resistant strains was found statistically significant (*p* = 0.02). Mutation in codon H526D was found in 3 isolates (4.61%, 1/50 in MDR and 2/15 in rifampicin mono-resistant isolates. One isolate (1.52%) each was found with mutation in codon D516V and codon H526Y in rifampicin mono-resistant isolates. On considering all isoniazid resistant isolates *katG* mutation was found in 46 isolates (56.09%, 32/50 in MDR and 14/32 in isoniazid mono-resistant isolates) whereas *inhA* mutation was found in 41 isolates (50%, 21/50 in MDR and 20/32 in isoniazid mono-resistant isolates). *katG*MUT1 (S315T1) mutation was found in 40 isolates (48.78%, 28/50 in MDR and 12/32 in isoniazid monoresistant isolates) and mutation in *katG*MUT2 (S315T2) was found in 3 isolates (3.67%, 2/50 in MDR and 1/32 in isoniazid monoresistant isolates) on comparing difference between MDR and isoniazid monoresistant isolates no statistically significant difference was seen for these two mutations. *InhA*MUT1 (C15T) mutation was found in 22 isolates (26.83%, 7/50 in MDR and 15/32 in isoniazid monoresistant isolates); *inhA*MUT1 difference in MDR isolates and isoniazid monoresistant isolates was statistically significant (*p* = 0.0010). Further mutation in *inhA*MUT3A was found in 1 (1.23%) MDR isolate. The detailed description of band patterns of drug resistant *Myco-bacterium tuberculosis* isolates by Genotype MTBDR *plus* assay is given in Table 1.

**Table 1.** Band patterns of drug resistant *Mycobacterium tuberculosis* isolates by Genotype MTBDR *plus* assay.

**Gene**	**Band**	**Gene region/Mutation**	**RIF mono resistant isolates (n=15)**	**INH mono resistant isolates (n=32)**	**MDR isolates (n=50)**	***p*-value RIF mono resistant Vs. MDR isolates; INH mono resistant Vs. MDR isolates**
*rpoB*	WT1	506–509	13 (86.70%)	32 (100%)	47 (94%)	0.35
	WT2	510–513	15 (100%)	32 (100%)	49 (98%)	-
	WT3	513–517	15 (100%)	32 (100%)	48 (96%)	-
	WT4	516–519	15 (100%)	32 (100%)	47 (94%)	-
	WT5	518–522	15 (100%)	32 (100%)	48 (96%)	-
	WT6	521–525	11 (73.33%)	32 (100%)	40 (80%)	0.58
	WT7	526–529	11 (73.33%)	32 (100%)	36 (72%)	0.92
	WT8	530–533	4 (26.27%)	32 (100%)	12 (24%)	0.83
	MUT 1	D516V	1 (6.69%)	0	0	-
	MUT2A	H526Y	1 (6.69%)	0	0	-
	MUT2B	H526D	2 (13.33%)	0	1 (2%)	0.07
	MUT3	S531L	3 (20.00%)	0	27 (54%)	0.02

*katG*	WT	315	15 (100%)	30 (93.75%)	25 (50%)	0.000039
	MUT 1	S315T1	0	12 (37.50%)	28 (56%)	0.10
	MUT 2	S315T2	0	1 (3.12%)	2 (4%)	0.84

*inhA*	WT1	−15/−16	15 (100%)	26 (81.25%)	45 (90%)	0.26
	WT2	−8	15 (100%)	27 (84.37%)	33 (66%)	0.07
	MUT 1	C15T	0	15 (46.87%)	7 (14%)	0.0010
	MUT2	A16G	0	0	0	-
	MUT3A	T8C	0	0	1 (2%)	-
	MUT3B	T8A	0	0	0	-

RIF = rifampicin; INH = isoniazid; MDR = multidrug resistant.

## DISCUSSION

Increasing trends of MDR-TB rates in high TB burden countries has emphasised the need of rapid diagnostic tests for accurate diagnosis of MDR-TB and has resulted in emergence of various rapid diagnostic tests for rapid diagnosis of MDR-TB. Among all available rapid diagnostic tests GeneXpert MTB/RIF is the most rapid test for diagnosis of MDR-TB but GenoType MTBDR *plus* assay offers an additional advantage of detection of isoniazid mono-resistant which cannot be detected by the GeneXpert MTB/RIF. Further studies in recent past (15, 16) has reported GenoType MTBDR *plus* assay as highly sensitive and specific test for rapid detection of MDR-TB; thus in the present GenoType MTBDR *plus* assay was preferred over GeneXpert MTB/RIF for detection of MDR-TB.

In the present study 37.89% (97/256, 15 RIF monoresistant, 32 INH monoresistant & 50 MDR) clinical isolates were found resistant to one or more drug which is almost comparable to our earlier study from this region where 33.5% isolates were found resistant to one or more drug (17), a study from Mysore district of Karnataka also reported 34.2% drug resistant isolates (18). Compared to our earlier study in which samples from entire district were included; in the present study samples were included from a single Tertiary care centre; Further in present study subjects suspected of MDR-TB were specifically recruited which might have resulted in slight higher percentage of total cases. MDR-TB is estimated in 3.6% new TB cases and 20.2% in previously treated cases globally (19). Studies from India have reported MDR-TB in 3.6–6.2% in new cases and 17.4%–53% in retreatment cases in the present study we found 19.53% MDR-TB cases (21.23%, 48/226 in previously treated cases, 6.67%, 2/30 in new cases) (18–21). Rifampicin resistance is associated with mutations in 81 base pair region (codon 527–533) of *rpoB* gene (22). In the present study rifampicin resistance was seen in 65 isolates (25.39%, 15 RIF monoresistant and 50 MDR) the most common mutation associated was found to be in *rpoB* S531L (30/65, 46.85%) which was in concordance to previous studies (19, 21, 22) from India but a statistically significant difference (p=0.02) was seen when MDR and rifampicin monoresistant isolates were compared, similar findings were also seen in studies from South Africa and Uganda (23, 24). Other mutations detected in *rpoB* codon D516V (1 isolate, 1.52%), *rpoB* codon H526Y (1 isolate, 1.52%) and *rpoB* codon H526D (2 isolates, 3.07%) were seen in rifampicin monoresistant strains only whereas *rpoB* codon H526D mutation was found in only 1 (1.52%) MDR isolate. Isolates with unknown mutations (missing Wild Type probe without any mutation bands) has been variably reported in earlier studies (25, 26). We also found 33 (50.77%) such isolates in the present study. Studies have reported high prevalence of *katG* mutation in high burden countries (27). In present study *katG* mutation in codon S315T1 was found in 48.78% isolates (12/32 in isoniazid mono resistant and 28/50 in MDR isolates.) whereas mutation in codon S315T2 was found in 3.65% isolates (1/32 in isoniazid monoresistant and 2/50 in MDR isolates), further *inhA* mutation in codon C15T was found in 26.83% isolates (15/32 in isoniazid mono-resistant and 7/50 in MDR isolates); mutation in codon C15T was more common in isoniazid monoresistant isolates as compared to MDR isolates (*p*= 0.0010), further one MDR isolate (1.22%) showed mutation in codon T8C. On considering unknown mutations (due to missing wild type) in *inhA* and *katG* gene, *katG* mutation was observed in 3.66% (3/82) isolate whereas *inhA* mutation was observed in 26.83% (22/82) isolates. Further previous studies have reported high prevalence of *katG* mutation than *inhA* gene mutation but we didn’t observe any significant difference between the two (22, 26).

Hetero-resistance (presence of all wild type probes along with one or more mutant bands) in MDR, rifampicin monoresistant and isoniazid monoresistant isolates was found to be 26% (13/50), 20% (3/15) and 34.37% (11/32) respectively. Authors in the past have also reported higher percentage of hetero-resistant isolates in their studies (21, 28). Eleven smear positive isolates were found to be NTM which highlights the utility of GenoType MTBDR *plus* assay over smear based diagnosis. One of the limitations of this study was inability to perform sequencing which might have provided better insight of hetero-resistant isolates.

GenoType MTBDR *plus* assay offers rapid detection of Rif and INH resistance, routine use of this assay for the diagnosis of pulmonary Tuberculosis can substantially reduce burden of tuberculosis by early detection of MDR-TB providing better management of patient. In the present study mutation in S531L codon in *rpoB* gene among rifampicin resistant isolates and mutation in codonS315T1 in *katG* gene and codon C15T in *inhA* gene among isoniazid resistant isolates were found most commonly associated with drug resistance. Further mutations in unknown regions and hetero-resistance was seen in very high number of MDR isolates thus; future studies based on sequencing are desirable in this region to fully explore the mutations associated with MDR-TB.
